# Planning fecal sludge management systems: Challenges observed in a small town in southern India^☆^

**DOI:** 10.1016/j.jenvman.2020.111811

**Published:** 2021-03-01

**Authors:** Reeba Devaraj, Rajiv K. Raman, Kavita Wankhade, Dhanik Narayan, Navamani Ramasamy, Teja Malladi

**Affiliations:** Indian Institute for Human Settlements, IIHS Chennai: Floor 7A, Chaitanya Exotica, 24/51 Venkatnarayana Road, T Nagar, Chennai, 600 017, India

**Keywords:** Urban sanitation, On-site systems, Fecal sludge management, India, Containment, de-sludging, SDG 6

## Abstract

Fecal Sludge Treatment or Septage Management is increasingly being recognised as an effective and appropriate method to scale urban sanitation systems to achieve safe sanitation, particularly in small towns and cities. As implementation progresses, data-based evidence is emerging, highlighting the challenges faced on the ground, and the requisite planning necessary to address them. This paper presents the findings, challenges and possible ways ahead from a study conducted to provide data for Fecal Sludge Management (FSM) planning for a small town in a state in southern India. With the objective of understanding the nature of containment structures and on-ground desludging practices, 8,001 households and 1,667 establishments were studied in Periyanaicken-Palayam (PNP), a non-sewered Town Panchayat in Coimbatore District, Tamil Nadu, to provide evidence for effective decision-making. The study showed wide variations in the sizing and design of the containment systems, which, when combined with the irregular frequency of desludging, has implications for FSM planning by municipal bodies. This study also highlights the methodological difficulties in studying containment systems, exposes a significant response bias given the limited understanding of containment systems within households, and spotlights the difficulty in physically verifying the reported data given the underground nature of these systems.

## Highlights

• Wastewater treatment, an SDG6 focus, requires broadening scope of urban sanitation.
• Methodological difficulties exist in data collection on containment systems.
• Containments design and construction deviations from norms impact sanitation chain.
• Methods and practices to standardise new, and retrofit old containments needed.
• FSM planning needs to be cogniscant of variations in containment systems.

## 1 Introduction

The Millennium Development Goals aimed to provide access to improved sanitation. Keeping with that, the Sustainable Development Goals (SDG), particularly SDG 6, retains the focus for access to safe and improved sanitation, but also refers to reducing the amount of untreated wastewater.^1^ This effectively means that sanitation targets for SDG 6 can only be met if the focus expands from just access to the full cycle of sanitation – access, conveyance, treatment and re-use. Fecal Sludge Treatment or Septage Management is increasingly being recognised as an effective and appropriate method to scale urban sanitation systems to achieve safe sanitation, particularly in small towns and cities. As implementation progresses, data-based evidence is emerging, highlighting the challenges faced on the ground, and the requisite planning necessary to address them. This paper presents the findings, challenges and possible ways ahead from a study conducted to provide data for Fecal Sludge Management (FSM) planning for a small town in a state in southern India. (see Table 1, Table 2)

**Table 1:** Distribution of arrangements for household sanitation – Periyanaicken-palayam Town Panchayat.

Type of Residential property	No. of HHs	Proportion of households with access to sanitation through					
		Individual Household Toilets	No Latrine in own house, but				
			Shared Toilet	PT/CT	Combination of PT/CT + Shared Toilet	OD	Combination of OD + PT/CT + toilet
Plotted Housing	6,980	79.6	10.5	7.3	0.7	1.0	0.9
Mixed use	644	83.4	13.0	2.1	0.8	0.2	0.5
Group Housing	336	83.3	11.6	2.4	2.7	0.0	0.0
Slum Housing	41	58.5	2.4	14.7	0.0	24.4	0.0
**All Properties**	**8,001**	**79.9**	**10.7**	**6.7**	**0.8**	**1.0**	**0.9**

*Source:* TNUSSP survey 2018, Households contacted N = 8,896, No consent = 895.

**Table 2:** Distribution of reported septic tanks with or without outlets.

	No outlet	Outlet (soak-pits/open drains, reed bed etc/)	Total
HOUSEHOLDS			
Reported septic tanks that were waterproof	491	34	525
Reported septic tanks that were not waterproof	4463	490	4953
No response/don't know			282
Total septic tanks			5760
**ESTABLISHMENTS**			
Reported septic tanks that were waterproof	69	9	78
Reported septic tanks that were not waterproof	264	23	287
No response/ Don't Know			12
Total septic tanks			377

Around 31 per cent of India's population is urban according (Census 2011), and Tamil Nadu, with an urban population of 34.9 million (48 per cent of the total state population), is one its most urbanised states. There are 8.9 million households in urban Tamil Nadu, out of which 4.2 million (48 per cent) depend on on-site systems. In Tamil Nadu, Urban Local Bodies (ULBs) are classified into Municipal Corporations, Municipalities and Town Panchayats, depending on the population and income of the ULBs.^2^ There are 12 Corporations, 124 Municipalities and 528 Town Panchayats, accounting for 43 per cent, 32 per cent and 25 per cent of urban population respectively.

Urban Tamil Nadu largely depends on on-site sanitation systems (OSS), as only 27 per cent of household toilets are connected to networked sewer systems. Around 42 per cent depend on OSS like septic tanks and improved pit latrines (Census 2011).^3^ This percentage goes up to 50 per cent in Town Panchayats which have lesser population (average population size of around 15,000), and considerably lesser staff strength in the urban local body.

The Government of Tamil Nadu (GoTN) has committed to the rapid scaling up of FSM as a complementary solution to networked sewer systems in corporations and larger municipalities, and as a standalone solution in smaller municipalities and Town Panchayats. As a step towards recognising the significance of FSM, the GoTN issued the Operative Guideline for Septage Management in 2014, and subsequently launched a programme for scaling FSM.

Given the limited examples globally of scaling FSM, the GoTN chose two non-sewered town panchayats – Periyanaicken-palayam (PNP) and Narasimhanaicken-palayam (NNP) – in Coimbatore, Tamil Nadu, to demonstrate FSM as an economical and effective means to scale sanitation across its 500+ town panchayats. Extensive studies were conducted across the full cycle of sanitation to understand field realities and provide inputs for effective FSM planning. This paper outlines the findings from one of the baseline studies.

Sanitation mapping was carried out in PNP (2018) to aid the preparation of an FSM plan and to serve as a management and monitoring support system. This involved a census of properties (residential, commercial and others) with data collected on the household sanitation arrangements including details of OSS, and desludging practices followed. The process of data collection and analysis raised issues of data robustness, analytical tolerance/sensitivity and the practicality of the methods used in FSM design and planning.

This paper revisits the survey data on containment and de-sludging practices to discuss the ramifications for planning towards FSM and meeting the goal of SDG 6. It presents findings from the ground and then discusses the wider implications of the same for sanitation design and planning. It focusses on two specific components of the sanitation chain – containment systems, and de-sludging practices.

### 1.1 Study area

PNP town panchayat located 17 km north of Coimbatore city, has an area of 9.38 sq. km,^4^ (GoTN,) with a population of 25,930 comprising 7,377 households (Census 2011). Almost 83 per cent of households in PNP have individual household toilets, and 14 per cent depend on public sanitary conveniences (PSCs). Open defecation is reported at 3 per cent. Septic tanks constitute 55 per cent of containments, and improved pits 9 per cent (Census, 2011). Around 19 per cent of households reported to be connected to the sewered network, but there is no sewered network in PNP. Therefore, the reported numbers for sewers are incorrect. Anecdotal evidence suggests that covered drains were mistakenly identified as sewers by surveyors across multiple cities during Census (2011).

There are 13 PSCs including public toilets and community toilets located across the town (TNUSSP, 2016). Households largely depend on private de-sludging operators who use trucks called ‘cesspool vehicles’ to empty the containments and to carry the septage. There are four private operators located in and around PNP with eight cesspool vehicles (TNUSSP, 2018A).

PNP has a Fecal Sludge Treatment Plant (FSTP) with a capacity to treat 25,000 L of fecal sludge per day or a volume equivalent to the capacity of four to six cess pool vehicles. The FSTP, which uses mechanised technology to treat fecal sludge, is meant to serve a cluster of town panchayats including PNP.

## 2 Objectives and methods

This study was undertaken to help the urban local body to design, plan and execute an effective fecal sludge management plan for the town. It targeted 100 per cent coverage of all households and establishments, complemented by a mapping exercise. Given this, the objectives of the study were:

● To understand access, containment and on-ground desludging practices to enable more effective planning.
● To prepare a GIS-linked database of properties (households and establishments) and help build a spatially explicit database of containments and networks for conveyance and access that could be updated/tracked over time.
● To provide spatial and non-spatial inputs for effective decision-making.
● To validate the appropriateness of the selected locations and sizing of the treatment systems.

### 2.1 Methods

The study conducted from February to May 2018, attempted to cover all households and establishments in PNP through door-to-door data collection using surveys. There were three parts to the survey: a detailed questionnaire administered to the households, an observation form filled by the field team, and a recording of geo-coordinates of households and establishments. In addition, a total station study using surveying equipment was conducted to map access roads and streets and collect details on their width.

Since this paper presents only a sub-set of findings – on containment and de-sludging – it primarily utilises the information gathered from the questionnaires such as demographic details, types of sanitation arrangements, containment details including reported data on dimensions of the underground structure, materials used, and desludging practices. Information was also collected on the location of the drinking water source with respect to the containment structure, to understand the potential health risks that can arise from contamination. For a few findings, the paper draws upon mapping done for properties and roads.

The questionnaire was pre-tested and based on inputs from the field. A few questions were added and modified before the questionnaire was finalised and transferred to an Android app. Enumerators were then trained on the Computer Assisted Personal Interview (CAPI) methodology and data capture process. In order to ensure data accuracy, quality controls and checks were in-built within the data collection application. Further, throughout the data collection process, the research agency followed field quality procedures and protocols including back checks and accompanied calls.^5^ In addition to real-time data monitoring, IIHS had a clear field monitoring plan in which a sample of households and establishments were visited by the team to validate the collected data.

All buildings including residential, commercial, industrial, institutional and mixed use were visited. A total of 11,924 buildings were listed in PNP. During listing, these buildings were further classified into three categories based on their current functioning status – occupied, unoccupied or locked.^6^ This classification was used to select only those buildings that were eligible for further data collection. Only occupied buildings with an owner or occupant were considered eligible. Of the occupied buildings that is, 11,013 buildings, consent to capture building level details was obtained for 10,937 buildings which included 8896 household units and 2,041 establishments. In order to capture unit level details, consent was further obtained from households and establishments. A total number of 8,001 households and 1,667 establishments consented to the survey.

More than half of the respondents were not owners, and the establishments were mostly of the mixed-use and commercial type. Public and semi-public structures and industrial goods establishments were the most common type.

Depending on the type of land use, the surveyed residential buildings were classified as either mixed use or standalone residential use. In PNP, the majority of buildings (92 per cent; N = 7,357) were standalone residential and 8 per cent belonged to the mixed-use category.^7^ Residential buildings were further sub-categorized as group housing, plotted housing and slum housing. Most of the residential buildings were plotted houses, followed by group houses and slum houses. Of the 644 mixed-use buildings, the majority were used for both residential and commercial purposes. Of the 1,667 establishments surveyed, mixed-use and commercial type constituted the majority, accounting for 51 per cent and 36 per cent respectively. The majority of establishments (92 per cent) employed between one and ten employees.

A major challenge during data collection was that several households were reluctant to give consent, due to rampant theft in the area. The nature of information that the survey intended to capture, especially on containments, also proved to be a challenge since the structures were located underground, and hence could increase response bias.

## 3 Findings

Findings from the study are presented across the sanitation chain including access, containment and collection.

### 3.1 Sanitation arrangements in households and establishments

**Households Arrangements for Sanitation:** Around 80 per cent of the surveyed households had access to individual household toilets, which approximately tallied with Census (2011) data that reported a total number of 7,377 households and reported 83 per cent of households with individual household toilets. A significant proportion (87 per cent) of reporting households were residents in plotted houses^8^ exclusively for residential use. Of this, 80 per cent reported access to sanitation facilities within their houses. About 11 per cent of households reported access to shared sanitation facilities.

Properties of mixed-use character, which accounted for 8 per cent, were the next significant category in the town. Access to sanitation facilities within the premises was comparatively higher at 83 per cent. Shared facilities were reported by about 13 per cent of these households. Group housing (multiple households residing in the same building)^9^ accounted for only 4 percent of households. In this category, 83 per cent reported access to sanitation facilities within their house, while about 11 per cent reported using shared facilities.

About 1 per cent of the households were residents in slums,^10^ of which only 59 per cent had access to sanitation facilities within their premises. A significant proportion of households in this category (17.1 per cent) reported access to shared and community facilities, and nearly a quarter (24.4 per cent) reported resorting to open defecation. It is to be noted that the number of properties where households were locked, or consent was not given was higher in slum areas, hence the percentage of property types may not be representative.

For households that did not have individual household toilets, this study examined the availability of space to build toilets. Of the households with no access to toilets within their premises (1,607), 1,004 or 62.6 per cent reported that they did not have sufficient space to build a toilet. Hence, availability of space is a key constraint to building individual toilets within own premises.

**Arrangement for Sanitation in Establishments:** Sixty-four per cent of the establishments (n = 1,064) in PNP had access to sanitation facilities within their premises. Most remaining establishments (around 34 per cent) depended on shared toilets outside their premises as well as public/community toilets. Establishments reporting this arrangement had an average staff strength of 1.8. A small proportion of establishments (about 2 per cent) had a staff strength of just one, who reported resorting to defecating in the open. While the establishments were bucketed into various categories such as manufacturing, socio-cultural, and commercial, there were no significant variations across categories. The only notable finding was that all manufacturing establishments had toilets in their premises. Out of 603 establishments without toilets in their premises, 93 or 15.4 per cent reported adequate space for construction of sanitation facilities within the premises. It was also attempted to collect disaggregated data for women employees, but the responses were inadequate to come to a conclusion.

In PNP, 36 per cent of the establishments did not provide access to sanitation facilities within the premises which caused inconveniences for employees and visitors. However, it is not easy to establish whether these establishments were in compliance of the bye-laws or not. For shops and commercial offices, the byelaws indicate one water closet for every 25 persons or part thereof exceeding 15 (including employees and customers). Further, it for female personnel, 1 water closet should be available for every 15 persons or part thereof exceeding 10. (GoTN, 2019). Adequacy of sanitation facilities is mandated based on employee strength in the establishment and an estimate of customers. Since the study of customer footfall was beyond the scope of this study, it is not possible to comment on the adequacy of sanitation facilities for establishments. Out of the 12 public sanitation facilities in PNP, only one was a public toilet (TNUSSP, 2018B).^11^ Absence of adequate public toilet facilities, and lack of toilets in establishment (even if in compliance with the law) indicates a need for examine regulatory provisioning and access if there is a need to plan and design sanitation supplementary facilities in a sustainable manner.

### 3.2 Containment structures: construction practices and impacts

**Design of the containment structure:** In order to understand the type of containment systems, households (n = 6,394) and establishments (1,064) were asked about the containment system their toilets were connected to. A small percentage of households (4.5 per cent) and a significant percentage of establishments (63 per cent) were unable to respond to this question due to a lack of knowledge. A significant proportion of households (90.1 per cent, n = 5,760) with individual household toilets (n = 6,394) reported septic tanks as their containment system, as did 377 (or 35.4 per cent) of 1,064 establishments with toilets within their premises. The next most significant containment structure noted was the single pit, reported by 5.3 per cent households and 1.4 per cent establishments.

Other studies and discussions with masons and building contractors in different parts of the state had indicated that containment systems reported as ‘septic tanks’ deviate significantly from key design standards, which could affect their performance.^12^ In most cases, it is surmised that these so-called ‘septic tanks’ actually behave like leach pits.

Thus, for the study in PNP, a series of discrete questions regarding wall material, roof material, plastering of wall and base, presence of partition, and number of chambers, were asked. This helped validate the responses on the type of containment system present in the premises and confirm if they were built according to standards.

Of the 5,760 households that reported their containment structures as septic tanks, only 525 (9.1 per cent) households reported the walls and base as being plastered with the possibility of achieving watertightness as is required for a well-functioning septic tank. Some structures reported as ‘pit latrines’ noted the wall and/or base as being plastered.

**Fig. 1:**
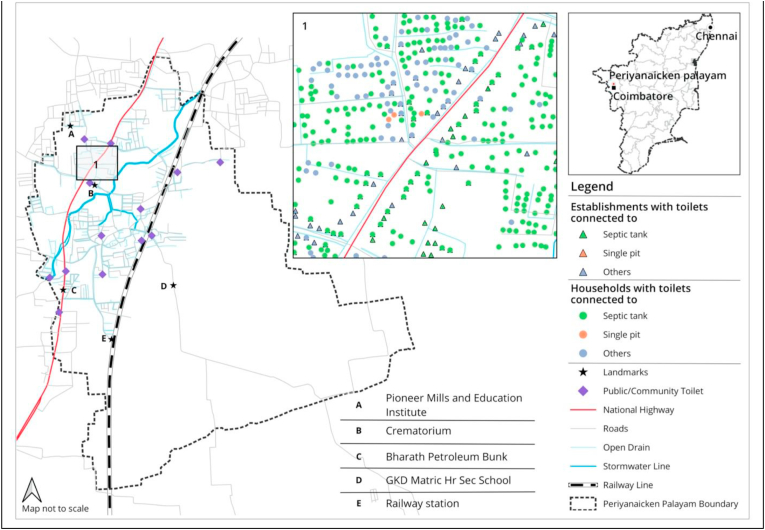
Map of Periyanaicken-palayam town panchayat.

**Fig. 2:**
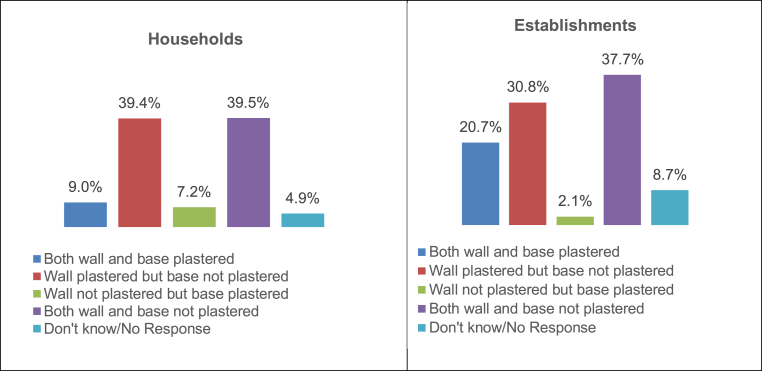
Coating for wall and base for reported ‘septic tanks'- Households and Establishments.

**Fig. 3:**
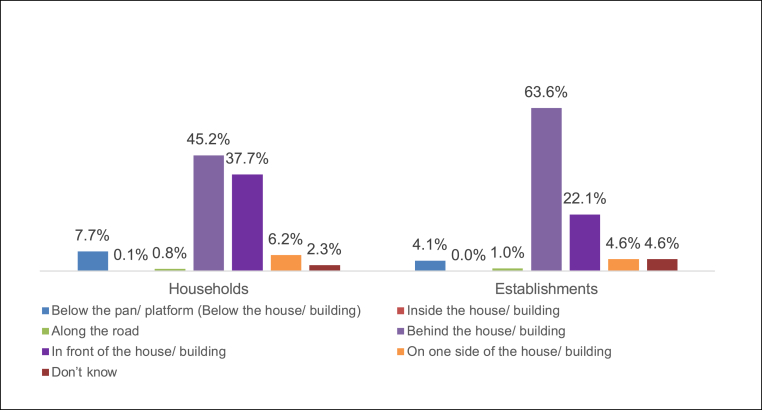
Location of OSS.

Fig. 2 details the coating practice for both wall and base materials in septic tanks reported by establishments (see Fig. 1). Only 78 (20.7 per cent) of the 377 septic tanks reported by establishments could possibly achieve water tightness, as they had both walls and base plastered (see Fig. 3).

Thus, amongst both households and establishments in PNP, the majority of the containments reported as septic tanks did not function in a water-tight manner, but worked in a manner similar to leach pits with either the wall or/and the base left without plastering.

**Partition:** In households, out of the 525 structures with walls and base plastered, 87 containments (16.5 per cent) were partitioned. Among establishments, out of reported 78 septic tanks with plastered walls and base, 17 (21.7 per cent) were partitioned. Most of the septic tanks in households and establishments with both walls and base plastered were single chamber tanks, and thus did not induce greater sedimentation or solid-liquid separation.

Out of 6,394 households and 1,064 establishments, only 1.5 per cent of households, and 4.5 per cent of establishments had partitions and were waterproof, thereby being able to function as a septic tank, as opposed to the reported percentages of 90 and 35 respectively.

**Disposal of septic tank effluents:** Households that reported having septic tanks were queried on practices followed for the disposal of effluents from the tank. In PNP, 90.7 per cent or 5,229 households reported that the containment structure did not have any outlet. Further, 7 per cent reported that the containment was connected to surface or open drains, and only 1.8 per cent of the households’ reported connecting containments to soak pits or leach pits. Amongst the establishments reporting septic tanks (n = 377), 90.5 per cent reported having no outlet and 10 per cent were connected to a soak-pit.

As reported earlier, many of these reported septic tanks had either walls or base left un-plastered. Therefore, in the absence of any outlet, it can be assumed that they function as leach pits. Out of 525 waterproof septic tanks in households, and 78 waterproof tanks in establishments, 93.5 per cent and 88.4 per cent respectively reported having no outlets. In these cases, the septic tanks functioned as holding tanks.

Overall, only 3 septic tanks in establishments were waterproof, had partitions and were connected to soak pits.

**Size of Containment structures:** Of 6,394 households with toilets within premises, 5,760 reported septic tanks, and 341 reported single pits. Out of these, 4023 households with reported septic tanks knew the size of their septic tanks, and 85 households with reported leach pits knew their dimensions. According to Indian standards, the recommended size of septic tanks for up to 5 users is 1,500 L, and the recommended size of single pits is 827 (CPHEEO, 2013). The mean household size of the reporting households was 3.22.

Most of the reported septic tanks in the households were over-sized, with nearly a third (34 per cent) reporting over-sizing by a factor of 10. About a quarter of the households (21 per cent) reported single pits with volumes that followed Central Public Health and Environmental Engineering Organisation (CPHEEO) recommendations, while the remaining were oversized. However, nearly 60 percent of the single pits were oversized by a factor of 2. It was observed that septic tanks had greater propensity to be oversized than pits, and significantly so. It is noted that many reported septic tanks functioned as pits, but since the size of pits requirements is even lesser, the over-estimation will only increase.

**Physical Verification:** Given that data on containment systems was heavily dependent on information provided by respondents, and that the subterranean nature meant minimal chances for observation, the study team carried out a physical verification of a sample of containment systems at households in PNP to cross check the responses given by households. The team physically measured containment dimensions and the measurements were then compared with the reported data. The team also checked for the presence of manholes and vent pipes.

Forty-three samples were checked. Given the lack of manholes as well as the reluctance of households to open the tank, only 4 were opened and observed. In the other 39 containments, the length and breadth were measures for only the visible portion of the septic tank on the top. The significant observations were as follows:

- Sample checks concluded that location and respondent names tallied.
- None of the containment structures had a separate soakaway.
- Manholes were not present for 75 per cent of checked samples.
- Length X breadth dimensions were verified and found to be within ± 20 per cent of reported data values.

**Vertical distance between containment systems and water source:** The maximum reported depth of single pits in PNP was 20 feet in households and 8 feet in establishments. Given that groundwater depth for PNP was over 90 feet (Water Resources Organisation, Tamil Nadu Public Works Department), the vertical safe distance between containments and water source had been maintained (Standards, n.d.).

**Accessibility of containment systems:** Direct and easy access to the containment system for desludging depends on three parameters: location of the containment system, ease of opening the lid, and width of the road to accommodate the desludging vehicles. This study covered the first two parameters and analysed these criteria for 6,101 households and 392 establishments who reported having either septic tanks or pit latrines.

Most households had containment systems that were located in accessible locations, and in a majority of cases, the structures were located either in front or behind the building. In a few households and establishments, the containment systems (reported septic tank/single pit) were located in a manner where access was challenging. In 469 (7.6 per cent) households and 16 establishments, (4.1 per cent) containments were located below the toilet pan/platform, or below the house or building, and hence were inaccessible.

Of 5,490 household containment structures that were located in an accessible area (either along the road, behind or in front of the house/building, or on one side of the house/building), 1,711 containments (31.12 per cent) had a manhole or pipe with cover. Likewise, in establishments, 29.8 per cent containments located in accessible areas had a manhole or pipe with cap, which are essential for inspection and desludging. The absence of a manhole would mean that the containment lid has to be broken in order to remove the fecal sludge. Accounting for inaccessible location and absence of cover, only 28 per cent of households and 27 per cent of establishments had accessible containment systems.

### 3.3 Emptying practices

Of the 6,101 households with reported septic tanks and single pits, only 8.2 per cent or 498 households reported having ever de-sludged their containment systems. Of the 5,760 reported septic tanks, only 8.2 per cent reported ever desludging. Further, of the 525 households that reported construction of septic tanks with walls and base plastered, only 22.8 per cent reported having ever de-sludged. Out of 87 households with reported wall and bases plastered and having partitions, 36.8 per cent or 32 households reported having ever de-sludged. Out of 491 households that had wall and bases plastered, and no outlet, 18 per cent reported ever having de-sludged. Only 29 establishments of the 393 reported having ever emptied the containment systems.

When the households and establishments who reported de-sludging at least once were asked about the interval of de-sludging, nearly one-third households and half the establishments reported de-sludging only when it fills up. Nearly a quarter of households and establishments reported that they had emptied the containment structure only once. Hence, only a few households and establishments emptied the tanks on a regular basis. This finding is not surprising since the earlier section showed that most containment systems actually performed as pit latrines.

### 3.4 Proximity of properties to treatment facility

Initial studies revealed that the lack of adequate treatment facilities within a certain distance was the primary reason for de-sludging operators to dump septage in the open. Hence, the government decided to increase number of treatment facilities across the state. There was a proposal to build an FSTP at PNP to service a cluster of FSTPs.

At the time of the study, the closest disposal facility for de-sludging operators was located in Coimbatore Municipal Corporation, at a distance of around 25 kms.^13^ A study had revealed that operators faced accessibility issues and restrictions from travelling on certain city roads during specific hours which made a huge impact on the business (TNUSSP, 2018A). Discussions with operators had highlighted that they would be willing to bring vehicles to the FSTP, provided that the distance from the households was not more than 10–12 km.

The purpose of this study was to also ascertain the suitability of the location of the proposed FSTP. Mapping showed that the closest distance from settlements in PNP to the FSTP was 2 kms and the farthest distance was 5 kms, implying that the FSTP site was at a suitable distance from the town panchayats.

## 4 Discussion

The findings show that considerable progress has been made in the provisioning of access to toilets. Only a small percentage of households practice open defecation, and need to be provided with individual, shared or community toilets. The key challenge in PNP (and illustrative of urban India) is to move households up the sanitation ladder. This is likely to be the case across much of urban India, where investments have been made in community toilets to provide access. There are three primary barriers in access to individual household toilets: lack of space, affordability and land tenure (WSP, 2016). This study has only explored the issue of land availability, and it appears to be a significant challenge given that two-thirds of households (1,004 or 63 per cent) did not have adequate space to build toilets. Out of 603 establishments without toilets in premises, 93 or 15.4 per cent reported adequate space for construction of sanitation facilities within the premises.

While there are certain methodological challenges that are discussed later, the findings clearly illustrate that there is widespread deviance from the standards in the construction of OSS. These include the absence of soakaways, oversized structures, and absence of openable lids. Amongst both households and establishments in PNP, the majority of the containments reported as septic tanks did not function in a water-tight manner, but instead were similar to leach pits with either the wall or/and the base, not plastered. Of the remaining septic tanks that could achieve watertightness, a large percentage did not have partitions. Thus, most of the reported septic tanks failed to function as septic tanks and instead behaved like leach pits, and in few cases, like holding tanks. Only a small percentage conformed to design specifications and could function as a septic tank. Other studies indicate that this could be because of lack of knowledge in the masons who constructed them, or because the households did not want to de-sludge frequently.

While the corrections for some deviances such as ensuring access to containment systems by putting a removable lid are easier to carry out, other design deviances are more complicated. For example, many structures reported as septic tanks did not have a soakaway, but most of these structures behaved like leach pits, and hence would not require a soakaway. Single pits could be an adequate solution in the interim if adequate distances are maintained from drinking water sources Containment systems require improvement, but rather than recommending the construction of new septic tanks, a more nuanced plan for retrofitting and improvement is required.

The findings highlight the need to pay attention to the containment systems, and this necessitates a two-pronged strategy: to ensure that new containment systems are built according to standards (BIS, 1985a, BIS, 1985b), and that existing ones are retrofitted or upgraded. One way to ensure that new containment systems are built to standards is by making necessary amendments to the building approval process, and ensuring that containment systems are verified before a building is approved. Towards this end, the Government of Tamil Nadu has passed amendments in the Building Rules to ensure that containment systems conform to standards and are verified in the building-plan approval stage (GoTN, 2019). This, however, is only the first step; processes for verification need to evolve and be developed as this moves forward.

This still leaves open the question of retrofitting existing OSS. A requisite containment improvement plan at the household level with technology options and low-cost improvements could be devised. While households might be reluctant to reconstruct their OSS, ULBs can opt for certain effective improvements such as ensuring manholes are built, which can be done without incurring a high cost. A rolling plan which allows the reconstruction of the containments as and when they fill up could also be devised.

It must be recognised that any containment improvement plan would require substantial time and effort for scaling. The immediate priority should be to safely secure the fecal sludge that is being emptied by ensuring proper conveyance and treatment. In addition, FSM planning for other parts of the chain – de-sludging and treatment – needs to account for the variance in containment systems, until they are retrofitted/upgraded.

Most guidelines that stipulate ideal de-sludging frequencies are based on the assumptions that containment systems are built to specifications, including meeting the size requirements. However, if the containment systems are larger, frequent de-sludging is likely to face resistance from households because it imposes an additional financial burden – something that ought to be taken into consideration, and to plan for affordable de-sludging. More importantly, it is not immediately clear that sticking to the stipulated 2-to-3-year cycle of de-sludging renders either the containment system or overall FSM planning safer. It is beyond the scope of this paper to comment on ideal de-sludging periods, but a case is being made by this paper to take local conditions into consideration before deciding de-sludging periods. It also calls upon a change in household behaviour because if even lengthier cycles are stipulated, the de-sludging should happen before the tanks overflow.

Further, variance in containment systems affects both the volume and nature of the fecal sludge. Treatment plants are usually designed on the basis of population served, and assuming a specific de-sludging interval could lead to over-estimation of sizes. Local data collection is imperative to address this. One of the practical ways to deal with uncertainties of volumes is to adopt a modular approach for treatment facilities like what is being done in Tamil Nadu, with land and capital kept in reserve for future expansion. While one can start small, it is imperative to build some redundancy, and secure financial flows in the medium term for expansion if needed.

The findings also highlight methodological challenges for gathering data. The paper demonstrates how there can be a ‘response bias' (Lavrakas, 2008)^14^ in household surveys about containments – where containment structures that are reported as ‘septic tanks’ may not function like septic tanks. About 90 per cent of households reported that they had septic tanks, but when further probed about the porosity of the structures as well as partitions, only 1.6 per cent of the structures met the criterion to be termed a septic tank. In addition, several households as well as a significant proportion of establishments did not know what type of containment system they had access to. Tenants are also unlikely to give accurate answers about de-sludging.

The respondent bias is due to multiple reasons: inadequate understanding amongst residents about technical terms used to describe containment systems; respondents not being in a position to observe the construction or repair of the containment; and a high proportion of tenants in the study area who may not know details about the construction. All these biases affect survey results, as well as estimations such as dimensions of containment systems and their functioning.

Data collection regarding containment systems is further complicated by thr fact that they are located underground. Data regarding the structures e.g., size and porosity of the structure, and presence of partitions is household reported data which the team tried to cross-verify through observation. However, only some features could be ‘observed’; for the other, the septic tanks need to be opened, and there was resistance to do so from households.

One of the ways to overcome/compensate this is to triangulate data collected at the property level (households and establishments) by gathering information from other stakeholders. Discussions with masons is likely to reveal local practices of construction, as well as a sense of dimensions. Further, to understand de-sludging frequency, information can be triangulated by speaking with de-sludging operators.

ULBs, who require such data for planning, may not be able to conduct a Census or detailed study. A simpler database could possibly be built through a sample study based on transects (Center for Applied Transect Studies, 1988)^15^.Information can be aggregated transect-wise and supplemented by primary data from interactions with local masons, builders or contractors. One could also start with a basic database of containment structures such as a simplified version of the current study and refine the database with data collected as and when each unit is de-sludged.

## 5 Conclusion

The learnings from the study point to a series of checks and steps that need to be taken to achieve and sustain SDG 6. Providing access to toilets, which government programmes such as Swacch Bharat Mission have kickstarted, and inculcating/ensuring their use which has been emphasised earlier as well, are only the beginning. The wider mandate of the SDGs, which includes the treatment of wastewater, requires practitioners, planners and administrators to broaden the scope of urban sanitation.

The above findings confirm what is generally accepted by practitioners based on anecdotal evidence – that there is significant deviance from prescribed standards in construction practices of OSS. Responses recorded as ‘septic tanks’ could actually be structures that behave as ‘holding tanks’ or ‘leach pits’. As the first link in the FSM chain, containments are a vital point of fecal disposal. Any variation in containment systems will have cascading implications for design and planning for subsequent parts of the chain. These findings are thus critical to design an appropriate scaling strategy for FSM.

This paper highlights the need to pay more attention to containment systems, a part of the chain often ignored. More work needs to be done to devise methods and practices to ensure that new containments are built to standard, and that old ones are retrofitted. Further, it is necessary to understand the implications of containment systems for FSM planning to avoid over-estimation of capacities.

This paper also points to a larger point that planning for relatively new areas like FSM must be grounded in the local context and realities. While thumb rules and standards are useful and can provide a baseline, it is essential to validate these with practices on the ground given local contexts. Also, any containment improvement plan will require engagement with households and establishments. Therefore, there is an increasing need to pay attention to the communication around FSM. The Kakkaman campaign in Tamil Nadu aims to address this by making sanitation communication fun (Nagarajan and Sudhakar, 2020).

Finally, this paper calls for increased attention to data collection methods to ensure effective planning. Large scale data sets like the Census in India are based on resident reported data, and hence are likely to be affected by respondent bias, in particular in the reporting of ‘septic tanks’. For instance, Census (2011) reports that 67 per cent of households in PNP are connected to septic tanks; but this study undertaken in PNP shows that in reality, most of these are not likely to function as septic tanks. A case could be made that large, generic data sets like those of the Census need to be supplemented by local data collection efforts for planning. There is a strong need to develop and implement methods for measurement which are simple and scalable, incorporating aspects of socio-economic nature of households/neighbourhoods and sub-surface character. While possibilities have been suggested in the Discussion section, this is an area that requires much research and innovation.

## Credit author statement

Reeba Devaraj: Writing – original draft, Formal analysis, Project administration, Data curation, Rajiv K Raman.: Conceptualization, Writing – review & editing, Supervision. Kavita Wankhade: Conceptualization, Writing – original draft, Writing – review & editing. Dhanik Narayan: Validation, Formal analysis.: Navamani Ramasamy: Project administration, Data curation, Validation.: Teja Malladi: Visualization, Validation.

## Competing interest

None.

## Declaration of competing interest

The authors declare that they have no known competing financial interests or personal relationships that could have appeared to influence the work reported in this paper.

## Footnotes

1 https://sdgs.un.org/goals/goal6.

2 Commissionerate of Municipal Administration (Accessed at https://www.tnurbantree.tn.gov.in/about-us/on 28 June 2020) and Directorate of Town Panchayat (Accessed at http://www.tn.gov.in/dtp/introduction.htm on 28 June 2020).

3 On-site sanitation system is a system in which excreta and wastewater are collected and stored or treated on the plot where they are generated. Containment systems are containers like a septic tank or a single pit in which the fecal waste from the toilet is stored. (Tilley et al., 2014).

4 Government of Tamil Nadu, P N Palayam Town Profile (Accessed at http://www.townpanchayat.in/Periyanaickenpalayam/town_profile on 28 Jun 2020).

5 Back checks – In-person visits conducted after the completion of an interview among randomly selected samples and by re-administering a few questions from the survey questionnaire to verify and validate the responses. Accompanied calls – The field supervisor is present during the entire duration of the interview as an observer to check on the quality of the interview.

6 Occupied building was any building that is under continuous or periodic habitation, occupancy or use. Unoccupied building was any vacant or under construction building that is not under continuous or periodic habitation, occupancy or use. Locked building was any building that is locked during the time of interviewer visit. The building maybe occupied or unoccupied. To distinguish between unoccupied and occupied, during listing, if they find a building locked, they will enquire nearby about the building and its status. If the building is in use but the owner is away or has been locked for a few weeks/months, the surveyor will visit the place two more times (not immediately but sometime during the study period). If the building is still locked even after two visits, the building is categorized as ‘locked'.

7 A mixed use building incorporates more than one uses into one structure such as residential, retail, hotel, entertainment, etc.

8 Plotted Development/Housing is a type of development layout wherein a stretch of developed land is divided into regular sized plots for uniform controlled building volumes (MoUD, 2016). This type of housing is common across different socio-economic classifications.

9 Group Housing refers to a building unit with one or more floors having more than two dwelling units, and having common service facilities where land is owned jointly (as in the case of co-operative societies or the public agencies, such as local authorities or housing boards, etc) (MoUD, 2016).

10 Slum Housing refers to houses located within slums. Slums are buildings that are in poor condition of maintenance or have compromised habitability due to poor ventilation, sanitation or otherwise are termed slums. These are generally declared or notified as slums under relevant legislation by competent authority. (MoUD, 2016).

11 The Advisory on Public and Community Toilets developed by Ministry of Housing and Urban Affairs, Government of India (2018) defines public toilets as facilities provided for floating population/general public in places such as markets, train stations or public areas and mostly used by undefined users (MoHUA, 2018).

12 Septic tanks are watertight single-storied tanks. The floor of the septic tank should be watertight and should be able to support the weight of the walls and contents. The walls maybe built of brick and should be plastered with cement mortar. For septic tanks that exceed 2,00 L, the tank may be divided into two chambers. The outlet should be connected to a seepage pit or dispersion trench from where it overflows into or is absorbed by the surrounding soil. The septic tank should be provided with a rectangle or circular opening for access (BIS, 1993). Leach pits/Twin pits should be lined to avoid collapsing. Bricks, stones or laterite bricks cement concrete rings could also be used depending upon their availability and cost. Except where precautions are to be taken to prevent pollution of water sources, the pit bottom should be left in a natural condition. RCC slabs should be used to cover the pit (CPHEEO, 2013). No standard exists in India for Ecosan, VIP and other OSS containment systems.

13 FSTP at PNP is operational currently, however was not operational at the time of the study.

14 Response bias is a general term that refers to conditions or factors that take place during the process of responding to surveys, affecting the way responses are provided. Such circumstances lead to a non-random deviation of the answers from their true value. (Encyclopedia of Survey Research Methods, Sage Research Methods, 2008) https://methods.sagepub.com/reference/encyclopedia-of-survey-research-methods.

15 A transect is a cut or path through part of the environment showing a range of different habitats. It is popularly used in biology and ecology. Centre for Applied Transect Studies. www.transect.org - Accessed on 25 June 2020.

## References

[bib2] BIS 2470 (1985). Indian Standard Code of Practice for Installation of Septic Tanks.

[bib3] BIS 2470 (1985). Code of Practice for Installation of Septic Tanks Part II- Secondary Treatment and Disposal of Septic Tank Effluent.

[bib5] Census (2011). Census of India. https://censusindia.gov.in/2011census/hlo/Data_sheet/India/Latrine.pdf.

[bib6] Center for applied transect studies, The Transect https://transect.org/transect.html (Accessed on 25 June 2020).

[bib7] CPHEEO (2013). Manual on Sewerage and Sewage Treatment.

[bib8] GoTN P. N palayam town profile. http://www.townpanchayat.in/Periyanaickenpalayam/town_profile%20on.

[bib9] GoTN (2019). Tamil Nadu Combined Development and Building Rules. https://www.chennaicorporation.gov.in/images/TNCDRBR-2019.pdf.

[bib10] Lavrakas P.J. (2008).

[bib11] MoHUA (2018). The Advisory on Public and Community Toilets.

[bib14] MoUD (2016). Model Building Bye-Laws.

[bib1] Nagarajan A., Sudhakar S. (2020). November 18). Let’s Talk Sanitation! Kakkaman Is Here. https://www.indiawaterportal.org/articles/lets-talk-sanitation-kakkaman-here-wtd2020.

[bib15] Tilley Elizabeth, Ulrich Lukas, Luthi Christoph, Reymond Philippe, Zurbrügg Christian (2014). Compendium of Sanitation Systems and Technologies.

[bib16] TNUSSP (2018 A). Desludging Operators in Periyanaicken-Palayam and Narasimhanaicken-Palayam - an Overview.

[bib17] TNUSSP (2018 B). Assessment of Community Toilets and Public Toilets in Periyanaicken-Palayam and NarasimhanaickenPalayam Town Panchayats of Coimbatore.

[bib18] TNUSSP (2016). City Sanitation Plan for Periyanaicken-Palayam.

[bib21] WSP (2016). Community Slum Sanitation in India: A Practitioners' Guide. https://www.wsp.org/sites/wsp/files/publications/Community%20Slum%20Sanitation%20in%20India.pdf.

